# Complete mitochondrial genome of the silver stripped skipper, *Leptalina unicolor* (Lepidoptera: Hesperiidae)

**DOI:** 10.1080/23802359.2019.1674725

**Published:** 2019-10-07

**Authors:** Su Yeon Jeong, Min Jee Kim, Na Ra Jeong, Iksoo Kim

**Affiliations:** Department of Applied Biology, College of Agriculture and Life Sciences, Chonnam National University, Gwangju, Republic of Korea

**Keywords:** *Leptalina unicolor*, mitochondrial genome, Hesperiidae

## Abstract

The silver stripped skipper, *Leptalina unicolor* Bremer and Grey, 1853 (Lepidoptera: Hesperiidae), is listed as endangered insect in South Korea. We sequenced the whole genome (15,854 bp) of *L. unicolor* species using Next-Generation Sequencing method and the subsequent gap-filling method. This genome included a set of typical genes and one major non-coding A + T-rich region, with an arrangement identical to that observed in most lepidopteran genomes. Twelve protein-coding genes (PCGs) had the typical ATN start codon, whereas *COI* had the atypical CGA codon that is frequently found in the start region of the lepidopteran *COI*. The 757-bp long A + T-rich region was the second largest among completely sequenced Hesperiidae, which ranged from 234 to 793 bp. Phylogenetic reconstruction was performed by maximum-likelihood method using the concatenated sequences of 13 PCGs and two rRNAs of available species of Hesperiidae, including that of *L. unicolor* (a total of 28 species). The resulting phylogeny provided strong support for monophyletic Heteropterinae in which *L. unicolor* belongs, with the highest nodal support and a sister relationship between current *L. unicolor* and co-subfamilial species *Carterocephalus silvicola* with a bootstrap value of 91%.

*Leptalina* is a monotypic temperate east Asian genus. The silver stripped skipper, *Leptalina unicolor* Bremer and Grey, 1853 (Lepidoptera: Hesperiidae), is distributed in Japan, eastern China, Russia Amur, and throughout Korea, except for the remote island of Jeju (Seok 1973). It occurs 2–3 times per year in the warmer region and once a year in the cold and mountainous region of Japan (Fukuda et al. 1984). In Korea, adults occur twice per year, showing seasonal dimorphism; the summer type adults produce more eggs than the spring type adults (Hong et al. 2016). In Korea, this species is under sudden decline in population (Ministry of Environment 2012) and, therefore, is listed as an Endangered species. *Leptalina unicolor* population decline has also been reported in Japan and Russia, and is listed as a Near Threatened species (Mano and Fujii 2009).

In 2016, an adult *L. unicolor* was collected from Inje-gun, Gangwon-do Province, South Korea (38° 01′ 39.92″ N, 128° 25 58.22″ E), and subsequently deposited as a voucher specimen at the Chonnam National University, Gwangju, Korea, under accession no. CNU7526. To sequence the mitogenome of *L. unicolor,* a library was prepared using the TruSeq Nano DNA Sample Preparation Kit. Whole genome sequencing was performed on the Illumina NextSeq-500 platform using a Mid Output v2 kit to produce 150 bp paired-end reads (Illumina, San Diego, CA, USA). Construction of the genome was conducted using MITObim ver. 1.9 (Hahn et al. 2013). Annotation of various genomic features was conducted using MITOS WebServer (http://mitos.bioinf.uni-leipzig.de/index.py) and subsequent manual BLAST search (Altschul et al. 1990). To fill and clarify gaps, a long fragment encompassing *ND5* and *lrRNA* genes was amplified and then short fragments (*CytB*∼*ND1*, *ND1,* and *ND1*∼*lrRNA*) were individually amplified using published primer sets (Kim et al. 2012). Phylogenetic analysis was performed using available species in the family Hesperiidae, including *L. unicolor*. Thirteen protein-coding genes (PCGs) and two rRNA genes of 30 mitogenome sequences, including those of the two outgroup species, were aligned and concatenated (12,487 bp including gaps). The substitution model, GTR + GAMMA + I, was selected using Modeltest ver. 3.7 (Posada and Crandall 1998). The maximum-likelihood (ML) method was conducted using RAxML-HPC2 on XSEDE ver. 8.0.24 (Stamatakis 2014), implemented on the CIPRES Portal ver. 3.1 (Miller et al. 2010). Trees were visualized with FigTree ver. 1.42 (http://tree.bio.ed.ac.uk/software/figtree/).

The complete 15,854-base pair (bp) mitogenome of *L. unicolor* is composed of typical gene sets (two rRNAs, 22 tRNAs, and 13 PCGs) and a major non-coding A + T-rich region (GenBank acc. no. MK265705). The length of A + T-rich region of *L. unicolor* is 757 bp, which is the second largest in Hesperiidae (793 bp in *C. silvicola*, Kim et al. 2014). The gene arrangement of the *L. unicolor* mitogenome is identical to that of the ditrysian Lepidoptera (Kim et al. 2010). However, cofamilial *Erynnis montanus* has been reported to have *trnS*-*trnN* arrangement at the tRNA cluster region located between *ND3* and *ND5* genes, instead of *trnN*-*trnS* arrangement (Wang et al. 2014). Twelve PCGs had the typical ATN start codon, whereas *COI* gene had an atypical CGA codon frequently found in the start region of the lepidopteran *COI* gene (Kim et al. 2012).

Phylogenetic analyses showed that Heteropterinae, which includes current *L. unicolor,* formed a strong monophyletic group (Bootstrap support [BS] = 100%). Likewise, Megathyminae (100%), Eudaminae (100%), Pyrginae (100%), and Coalidinae (100%) were strongly supported as monophyletic groups, whereas Hesperiinae and Pyrginae were non-monophyletic groups (Figure 1). *Lerema accius* and *Parnara guttata* in Hesperiinae grouped together with Megathyminae, forming a strongly supported group (99%). *Euschemon rafflesia* in Pyrginae placed as the sister to all subfamilies, except for Coalidinaeo, whereas the other two co-subfamilial species, *E. montanus* and *Pyrgus maculatus* in Pyrginae, formed a strong monophyletic group. A previous mitogenome-based phylogeny showed that *Potanthus flavus*, *L. accius*, and *P. guttata* in Hesperiinae formed a strong monophyletic group (Zhang, Cong, Shen, et al. 2017). This result is inconsistent with the present results, indicating the need for further in-depth analysis and taxon sampling. The placement of *E. rafflesia* as a sister to the rest of Hesperiidae subfamilies, except for Coeliadinae, was also supported in previous mitogenome-based phylogeny (Zhang, Cong, Shen, et al. 2017).

**Figure 1.**
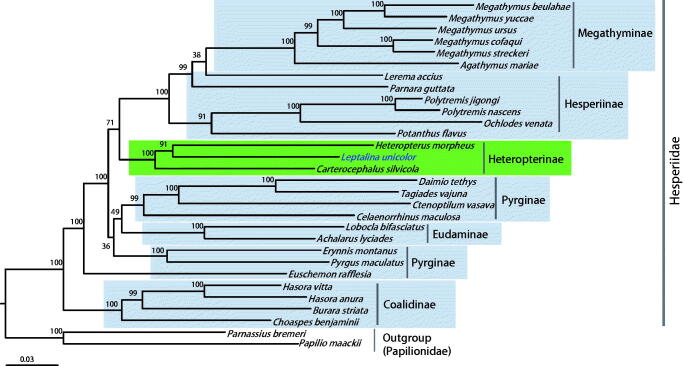
Phylogenetic tree of Hesperiidae, using maximum-likelihood (ML) method based on concatenated 13 protein-coding genes (PCGs) and 2 rRNAs. The numbers at each node specify bootstrap percentages of 1000 pseudoreplicates. Papilionidae (*Papilio maakii* and *Parnassius bremeri*) were utilized as the outgroup. GenBank accession numbers are as follows: *Pyrgus maculatus*, KP689265 (unpublished); *Euschemon rafflesia*, KY513288 (Zhang, Cong, Shen, et al. 2017); *Tagiades vajuna*, KX865091 (Liu et al. 2017); *Erynnis montanus*, KC659955 (Wang et al. 2014); *Celaenorrhinus maculosa*, KF543077 (Wang et al. 2015); *Daimio tethys*, KJ813807 (Zuo et al. 2016); *Ctenoptilum vasava*, JF713818 (Hao et al. 2012); *Achalarus lyciades*, KX249739 (unpublished); *Lobocla bifasciatus*, KJ629166 (Kim et al. 2014); *Heteropterus morpheus*, KF881050 (unpublished); *Carterocephalus silvicola*, KJ629163 (Kim et al. 2014); *Polytremis jigongi*, KP765762 (unpublished); *Parnara guttata*, JX101619 (unpublished); *Potanthus flavus*, KJ629167 (Kim et al. 2014); *Lerema accius*, KT598278 (Cong and Grishin 2016); *Ochlodes venata*, HM243593 (unpublished); *Polytremis nascens*, KM981865 (Jiang et al. 2016); *Choaspes benjaminii*, JX101620 (unpublished); *Hasora anura*, KR189008 (unpublished); *Hasora vitta*, KR076553 (unpublished); *Burara striata*, KY524446 (Zhang, Cong, Fan, et al. 2017); *Megathymus yuccae*, KY630500 (Zhang, Cong, Fan, et al. 2017); *M. streckeri*, KY630501 (Zhang, Cong, Fan, et al. 2017); *M. ursus*, KY630502 (Zhang, Cong, Fan, et al. 2017); *M. cofaqui*, KY630503 (Zhang, Cong, Fan, et al. 2017); *Agathymus mariae*, KY630504 (Zhang, Cong, Fan, et al. 2017); *M. beulahae*, KY630505 (Zhang, Cong, Fan, et al. 2017); *Parnassius bremeri* FJ871125 (Kim et al. 2009); and *Papilio maackii* KC433408 (Dong et al. 2013).
